# Interleukin-37 in respiratory diseases: molecular mechanisms and immune modulation

**DOI:** 10.3389/fimmu.2025.1675791

**Published:** 2025-11-10

**Authors:** Jing Cao, Kun He, Zixiao Chen, Haibo Xu, Jiaona Wei, Xixin Yan, Beibei Song

**Affiliations:** The First Department of Pulmonary and Critical Care Medicine, The Second Hospital of Hebei Medical University, Hebei Key Laboratory of Respiratory Critical Care Medicine, Hebei Institute of Respiratory Diseases, Shijiazhuang, Hebei, China

**Keywords:** interleukin-37, asthma, pulmonary infection, NSCLC, pulmonary interstitial fibrosis

## Abstract

Interleukin-37 (IL-37) is a potent anti-inflammatory cytokine that plays a crucial protective role in cancer, autoimmune diseases, and inflammatory diseases though its unique dual intracellular and extracellular action pathways. This review highlights the significance of IL-37 in common respiratory diseases. Specifically, IL-37 can alleviate asthma by inhibiting Th2/Th17 immune responses, inhibiting the release of epithelial-derived alarmins (TSLP and IL-33), and attenuating airway remodeling. In pulmonary infections, IL-37 modulates host responses by mitigating virus-induced hyperinflammation and inhibiting viral replication, as observed in COVID-19 and influenza, while also regulating immunopathology in Mycobacterium tuberculosis and fungal infections. Moreover, in non-small cell lung cancer (NSCLC), IL-37 directly suppresses tumor proliferation and migration, and restrains tumor progression through immunomodulation and angiogenesis regulation. In pulmonary fibrosis, IL-37 reduces collagen deposition and promotes autophagy, thereby counteracting interstitial fibrosis. Collectively, these findings demonstrate that IL-37 serves as a crucial immunomodulator in respiratory diseases, and targeting IL-37 offers novel insights and strategic opportunities for clinical intervention. This review systematically summarizes the molecular mechanisms of IL-37 and discusses its clinical therapeutic potential.

## 1 Introduction

Asthma, lung tumors, pulmonary fibrosis, and lung infections caused by viruses and bacteria, such as severe acute respiratory syndrome coronavirus 2 (SARS-CoV-2), are all significant global health concerns that can result in substantial morbidity and mortality (1, 2). As the body’s bridge to the outside world, the lungs frequently experience external insults that threaten host homeostasis. Inflammatory damage and immune dysregulation are the main pathogenic mechanisms for respiratory diseases (3). Current drug therapy focuses on lowering lung inflammation and airway obstruction to control symptoms and slow disease progression (4). However, existing drugs suffer from various side effects and lack adequate therapeutic efficacy. Therefore, significant efforts have been undertaken to develop new pharmacologic strategies for treating respiratory diseases (5).

IL-37 gene was first identified in 2000 by in silico studies of gene databases (6). IL-37 is a new anti-inflammatory cytokine having immunomodulatory properties, in contrast to the pro-inflammatory cytokines of the IL-1 superfamily (7). The eleven pro-inflammatory IL-1 family members are IL-36γ, IL-33, IL-36β, IL-18, IL-36α, IL-1α, and IL-1β. Although IL-37 and IL-38 are anti-inflammatory factors, IL-1Ra and IL-36Ra are receptor antagonists (8, 9). IL-37 and IL-1Ra possess a stable β-barrel structure that binds to the extracellular immunoglobulin-like domains of IL-18Rα and IL-1R1, respectively (2–6). Other cytokines in the IL-1 family have different activities, although they all have the same β-trefoil secondary structure (10–12). This is because signaling downstream of this receptor is controlled in different ways.

Recent years have witnessed significant progress toward comprehending IL-37 structure and its signaling pathways (13). Unlike conventional cytokines, which typically operate through a singular extracellular signaling paradigm—engaging cell-surface receptors to initiate downstream cascades that either amplify or suppress inflammation—IL-37 exerts its regulatory effects via a bimodal signaling mechanism that encompasses both intracellular and extracellular pathways. Several human diseases have been associated with abnormal IL-37 expression. IL-37 is a potential therapeutic target due to its protective function in the pathogenesis and progression of metabolic disorders, cancer, and inflammatory and autoimmune diseases (14–17). However, IL-37 can also act as a common contributor to cancer and chronic infection immunosuppression. It leads to abnormal cytokine proliferation and aberrant immune responses, exacerbating infection and inflammation (18, 19). These findings suggest that IL-37 may help maintain immune system homeostasis.

This review summarizes the effects of IL-37 on respiratory diseases, including bronchial asthma, coronavirus disease of 2019 (COVID-19), tuberculosis, fungus, lung cancer, and pulmonary fibrosis. Furthermore, the advancement of IL-37 research in respiratory diseases was assessed. Finally, the therapeutic potential of IL-37 in respiratory illnesses was revealed.

## 2 Introduction to IL-37

### 2.1 Discovery and subunit composition of IL-37

Three different research groups found IL-37 in 2000 after analyzing the human EST (expressed sequence tag) database (20–22). IL-37 was initially known as IL-1H, IL-1F7, IL-1RP1, and IL-1H4. It wasn’t until 2010 that Nold et al. (6) consolidated the nomenclature to IL-37, a designation that has persisted. The human IL-37 gene is situated in the 2q12–13 region on the long arm of chromosome 2, spanning 3.617kb, and is in close proximity to the regulatory regions of the IL-1α and IL-1β genes (23), within a gene cluster that encompasses multiple IL-1 family members, excluding IL-18 and IL-33. This unique genomic positioning may be fundamental to its distinctive anti-inflammatory characteristics (24, 25).

The IL-37 gene is composed of 6 exons, with exons 4, 5, and 6 encoding the β-trefoil structure that defines its extracellular function (8). The gene’s alternative splicing yields five isoforms: IL-37a, b, c, d, and e (7). IL-37a includes the N-terminus encoded by exon 3 and forms the β-trefoil with exons 4 to 6; IL-37b is the full-length, functionally rich isoform, with its β-trefoil encoded by exons 4 to 6; IL-37c and e lack exon 4 and are incapable of forming the β-trefoil, thus devoid of cytokine function; IL-37d possesses a complete β-trefoil and retains cytokine activity. Current research is primarily focused on IL-37b and d (23).

The IL-37b isoform can form homodimers, consisting of two head-to-head symmetrical β-trefoil units (24, 25). However, this dimeric form tends to diminish the anti-inflammatory efficacy of IL-37, serving as a negative regulatory factor of its activity (18). In contrast, the monomeric form of IL-37 is more effective in suppressing innate immunity (25, 26), highlighting the potential significance of developing an efficient monomeric IL-37b for the treatment of inflammatory and immune-mediated diseases.

### 2.2 Distribution and expression of IL-37

IL-37 is extensively distributed across a variety of human tissues and organs, with different isoforms exhibiting distinct expression levels in various locations (27–29). IL-37a is predominantly found in lymph nodes, placenta, colon, lungs, testes, and brain; IL-37b is present in lymph nodes, blood, placenta, colon, lungs, and testes; IL-37c is detected in lymph nodes, placenta, colon, lungs, testes, and kidneys; IL-37d is specifically expressed in testes, blood monocytes, bone marrow, umbilical cord mesenchymal stem cells, and adipose-derived stromal cells; IL-37e is limited to testes and bone marrow (30). IL-37 is produced by a diverse array of cell types, including activated B cells, monocytes, keratinocytes, endothelial cells, epithelial cells, dendritic cells (DCs), macrophages, CD4+ Treg cells, and plasma cells, and can be identified in both normal and malignant tissues (6, 31, 32).

Despite its broad distribution, IL-37 is expressed at low levels under physiological conditions, a trait attributed to the presence of unstable regions and a short half-life in IL-37 mRNA (33). Serum IL-37 concentrations in healthy individuals are typically below 100 pg/mL (20), and IL-37 transgenic mice also exhibit very low or undetectable constitutive expression levels (34). However, pro-inflammatory cytokines such as TNFα, IFN-γ, and IL-1β can upregulate IL-37 expression (6), while IL-12, IL-32, and GM-CSF can restrict IL-37 production (29, 33). Although basal IL-37 expression levels are low, its induced expression exerts significant anti-inflammatory and immune-modulatory effects (35). Abnormal IL-37 expression is observed in a variety of diseases, including autoimmune diseases (36–38), cardiovascular diseases (39, 40), neurological disorders (41, 42), liver disorders (43), skin diseases (13), asthma (44–46), infections (47, 48), and cancer (49).

### 2.3 Intranuclear actions of IL-37 and its pathways

Upon inflammatory stimulation, the expression of IL-37 increases within cells (46). During this process, the immature pro-IL-37 undergoes cleavage mediated by caspase-1, transforming into its mature form (50). This transition is crucial for IL-37’s translocation into the nucleus, as the use of caspase-1 inhibitors has been shown to significantly reduce IL-37’s ability to enter the nucleus (51, 52). While caspase-1 plays a pivotal role in the maturation of IL-37, other enzymes may also be involved in this cleavage process, indicating that the processing of IL-37 may occur through multiple pathways (53).

The mature IL-37 then forms a complex with the signal transducer protein Smad3, known as the IL-37-Smad3 complex, which can enter the cell nucleus and function as a transcription factor, regulating the transcriptional activity of pro-inflammatory genes (54). The nuclear translocation and functional execution of IL-37 are regulated by cleavage sites; mutations in these sites can impede the nuclear translocation of IL-37, subsequently reducing the expression levels of inflammatory factors (52, 55).

Once the IL-37/Smad3 complex successfully enters the nucleus, it can promote the production of protein tyrosine phosphatase non-receptor type (PTPNs), an enzyme that inhibits the activity of various inflammatory factors, such as Tumor Necrosis Factor-alpha (TNF-α) and IL-6. Additionally, PTPNs can modulate multiple inflammation-related signaling pathways, including MAPK subfamilies (p38, JNK, ERK), PI3K/Akt, NF-κB, and JAK-STAT pathways (56, 57). Through these mechanisms, PTPNs contribute to the suppression of inflammatory responses, thereby demonstrating the anti-inflammatory characteristics of IL-37.

### 2.4 Extracellular actions of IL-37 and its pathways

Secreted IL-37, acting as a ligand, binds to the interleukin-18 receptor alpha (IL-18Rα) (46). This interaction further recruits the interleukin-1 receptor 8 (IL-1R8), forming an IL-37/IL-18Rα/IL-1R8 complex (58). IL-1R8, as a negative regulatory factor, helps to suppress inflammation induced by IL-1 and IL-18 (59).

The anti-inflammatory effects of IL-37 are partly dependent on the presence of IL-1R8. Experiments have indicated that IL-1R8 is necessary for IL-37 to exert its anti-inflammatory actions, as the protective effects of IL-37 are diminished in the absence of IL-1R8. Interestingly, a recent study found that high-dose of IL-37 instead inhibit the recruitment of IL-1R8 and preferentially bind to IL-18Rα, a mechanism that ultimately limits the anti-inflammatory activity of IL-37 in macrophages (60). Furthermore, IL-37 can induce the ubiquitination and degradation of IL-1R8 through glycogen synthase kinase 3β (GSK3β), suggesting a complex regulatory mechanism between IL-37 and IL-1R8 (61).

Although IL-37 shares sequence homology with IL-18, it does not directly antagonize IL-18 (53). IL-18 binding protein (IL-18BP) is an IL-18 antagonist. The affinity of IL-18BP (400 PM) for IL-18 is higher than that of IL-18Rα. IL-18BP inhibits IL-18 binding to IL-18Rβ and IL-18Rɑ, resulting in anti-inflammatory effects (62). IL-37 indirectly regulates the activity of IL-18 by binding non-competitively to IL-18Rα and interacting with the IL-18BP (63, 64). This interaction may affect the anti-inflammatory effects of IL-37, as the binding of IL-18BP to IL-37 reduces its availability, thus weakening its anti-inflammatory action (22, 53).

**Figure 1:**
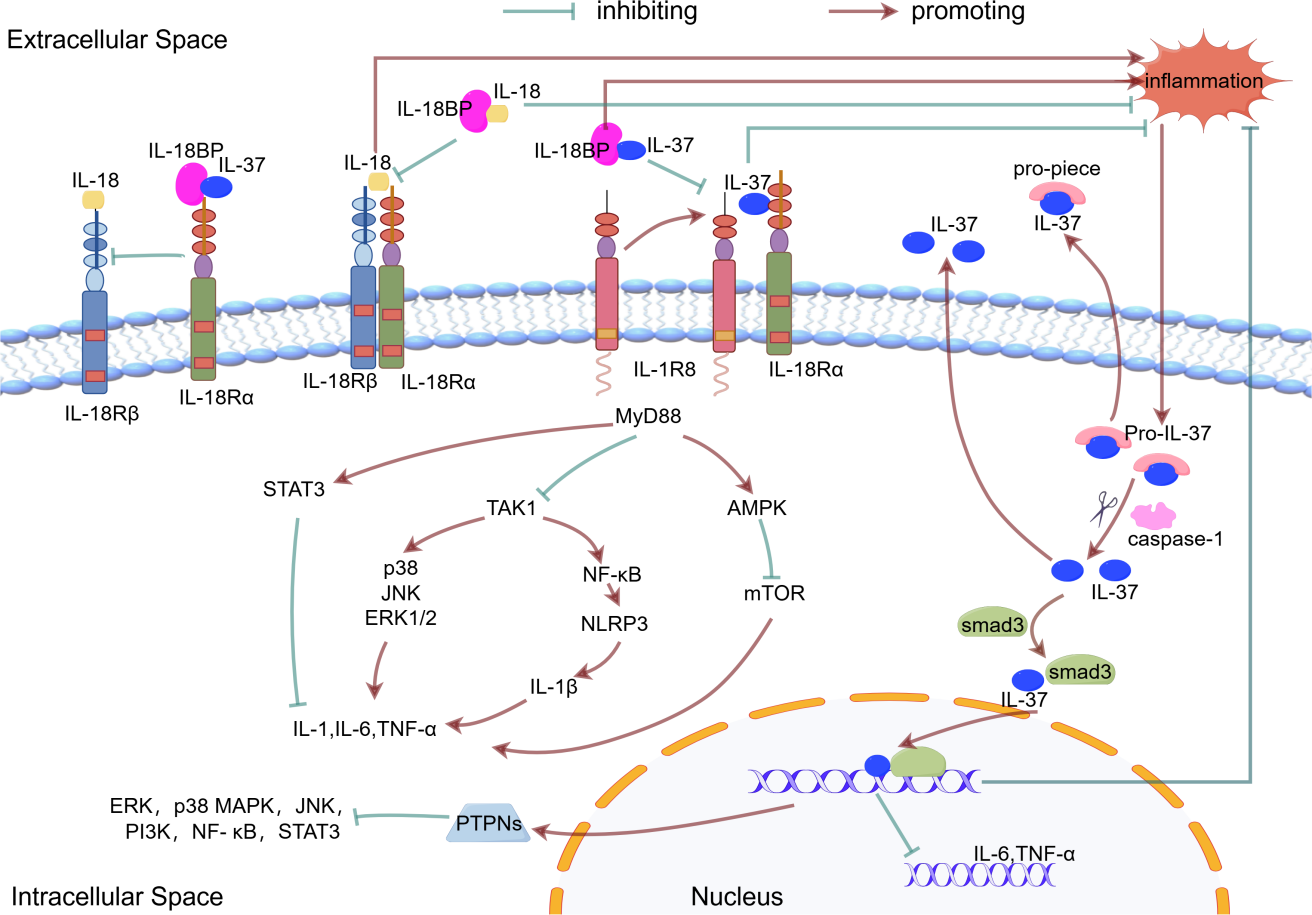
The mechanism of action of IL-37. Inflammatory stimuli increase the production of the precursor form of IL-37. This precursor is processed into mature IL-37 by caspase1. The mature IL-37 interacts with phosphorylated activated Smad3 in the cytoplasm, which contributes to nuclear translocation and gene transcription regulation. Both the precursor and mature forms of IL-37 are released from cells via non-classical secretion pathways. IL-37 binds to IL18Ra or IL18BP, which enhances the inhibition of IL18 and reduces the inflammatory response. IL-37 binds to IL-18Rα and recruits IL-1R8. This complex mediates the extracellular function of IL-37.

After binding to its receptor, IL-37 suppresses downstream signaling hubs MyD88 (in TLR pathways) (65, 66) and TAK1 kinase, leading to broad inhibition of downstream pro-inflammatory components - including transcription factors (NF-κB), kinase cascades (JNK, ERK, Fyn/Src), metabolic regulators (mTOR), and inflammasomes (NLRP3) - which collectively reduce pro-inflammatory effector molecules (IL-1β, TNF-α, IL-6, IL-8, IL-17) (41, 46, 57), thereby coordinately reducing the activation and infiltration of inflammatory cells and alleviating inflammatory responses. (**Figure 1**).

### 2.5 Biological functions of IL-37

No mouse or chimpanzee IL-37 homologs have been detected, unlike other IL-1 family members (67). Thus, most IL-37 research has focused on treating IL-37tg mice and rIL-37 protein in mouse models. Compared with the research conducted using simulated transgenic cells, the use of IL-37 transgenic mice can more accurately reflect the role of this cytokine in the physiological environment of the body in relation to diseases (68). As mentioned earlier, IL-37 reduces pro-inflammatory chemokine secretion and suppresses acquired and innate immune responses via extracellular and intracellular pathways (46).

It effectively reduces the production of key pro-inflammatory cytokines such as TNF-α, Interleukin-1 beta (IL-1β) and Interleukin-6 (IL-6), thereby directly alleviating inflammatory conditions (56, 69). This notion has been also supported by a recent multi-omics study, which revealed that IL-37 levels are negatively correlated with pro-inflammatory markers (such as IL-6, IL-8, and CCL28) but positively correlated with the anti-inflammatory marker TGF-β1 (69).

Additionally, IL-37 regulates polarization state of macrophages and the function. *In vitro* experiments have shown that rIL-37 downregulates the expression of iNOS, CD11c, MCP-1, CD86, and IL-6 in M1 macrophages, while upregulating the expression of IL-10 and CD206 in M2 macrophages (60, 70, 71). The mechanism involves inhibition of the Notch1 and NF-κB pathways, thereby suppressing macrophage polarization toward the M1 phenotype (71). Therefore, IL-37 can shift macrophage polarization from the pro-inflammatory M1 phenotype to the anti-inflammatory M2 phenotype. Furthermore, IL-37 exhibits significant anti-inflammatory effects on macrophages by activating the PTEN/STAT3/AMPK signaling pathway, while inhibiting the AKT/Erk/NF-κB/mTOR pathway, and also suppressing the release of pro-inflammatory cytokines such as TNF-α, IL-6, and IL-1β. However, recent studies have revealed a dual role of IL-37 in macrophage inflammatory responses: at low doses, it exerts anti-inflammatory effects, whereas at high doses, it preferentially binds to IL-18R, inhibits the activation of PTEN, AMPK, and STAT3, and thereby stimulates pro-inflammatory activity in macrophages (60). To date, this phenomenon has only been observed in studies related to type 2 diabetes mellitus (T2DM). Therefore, further experiments are needed to validate the appropriate dosage of IL-37 in the context of specific diseases.

IL-37 also influences the maturation process of DCs, preventing their maturation and fostering the generation of antigen-specific regulatory T cells (Tregs), which are crucial for maintaining immune tolerance and inhibiting excessive immune responses (72–74). By reducing the expression of co-stimulatory molecules on the surface of DCs, IL-37 further diminishes T cell-mediated inflammatory reactions (17, 30). Furthermore, the immunosuppressive function of IL-37 is also associated with the regulation of cellular metabolism. Based on metabolomic analysis, Teufel et al. found that IL-37 reduces immune cell activation by modulating FGF-21, glutathione, glutamate metabolism, and phospholipid metabolism, as well as regulating vascularization (such as VEGFA) and ribosomal and protein translational activity (69). Moreover, IL-37 enhances its anti-inflammatory effects by increasing the expression of the anti-inflammatory cytokine IL-10, achieving a balance between pro- and anti-inflammatory cytokines in immune responses (34, 75). These combined actions endow IL-37 with significant therapeutic potential in the treatment of various inflammatory diseases.

In addition to its well-documented anti-inflammatory effects, emerging evidence indicates that IL-37 is also involved in the regulation of autophagy and apoptosis. Cao et al. (76) demonstrated that IL-37 not only ameliorates PM2.5-induced acute lung injury through its anti-inflammatory properties, but also significantly suppresses the upregulation of autophagy-related proteins (such as Beclin-1, ATG5, and LC3BII) and apoptosis-related proteins (including Bax and Cleaved Caspase-3) following PM2.5 stimulation. Further mechanistic investigation suggested that IL-37 may inhibit PM2.5-induced autophagy and apoptosis by activating the AKT/mTOR signaling pathway, which is known to suppress autophagy. Notably, Li et al. (77) previously reported that in hepatocytes, IL-37 induces autophagy through inhibition of the PI3K/AKT/mTOR pathway. Moreover, Zhang et al. (78) confirmed that IL-37 alleviates high glucose-induced podocyte injury—including inflammation, oxidative stress, and apoptosis—by suppressing the activation of the STAT3/CypA signaling pathway. These findings suggest that the regulatory effects of IL-37 on autophagy and apoptosis may be tissue- or context-dependent. The underlying molecular mechanisms warrant further in-depth and systematic investigation across various disease models, which may provide new theoretical insights and potential therapeutic targets for related diseases.

In addition to the above functions, IL-37 also plays a significant role in regulating angiogenesis, with its effects demonstrating complexity and duality. Sometimes it promotes angiogenesis, while at other times it inhibits it. *In vitro* experiments have shown that IL-37 can significantly promote the migration, proliferation, and tube formation of human umbilical vein endothelial cells. Further *in vivo* studies have also confirmed that IL-37 can promote both pathological and physiological angiogenesis in mouse models of ischemic retinopathy and Matrigel plug models (79). The specific mechanism of its pro-angiogenic effect may involve the transforming TGF-β signaling pathway (80). On the other hand, IL-37 has also been shown to have an inhibitory effect on angiogenesis, especially in the context of tumor treatment. In mouse orthotopic models of hepatocellular carcinoma (HCC) and diethylnitrosamine-induced HCC models, IL-37 can inhibit the development of liver cancer by suppressing tumor angiogenesis (81). In NSCLC, tumor cells transfected with IL-37 show reduced CD34 expression and decreased microvessel density (82). These results suggest that IL-37 may play an anti-tumor angiogenic role, thereby blocking the nutrient supply to tumors and inhibiting their growth and metastasis. This complex role of IL-37 may be related to its microenvironment; it mainly exerts an inhibitory effect in the tumor microenvironment, but tends to promote angiogenesis under specific pathological conditions such as hypoxia (79).

In the context of respiratory diseases, IL-37 plays a pivotal role, utilizing its anti-inflammatory and immune-modulating capabilities to effectively alleviate asthma symptoms (83, 84), reduce inflammation caused by infections (85), inhibit the growth of lung cancer (82), and mitigate the progression of pulmonary fibrosis (86, 87). These attributes highlight the substantial promise of IL-37 in the treatment of a range of respiratory conditions.

## 3 Association between IL-37 and respiratory diseases

### 3.1 Asthma

A common, long-term, inflammatory, and allergic respiratory condition, asthma is characterized by bronchospasm and reversible airway blockage (88, 89). The heterogeneity of asthma is an important aspect of the disease. Different asthmatic patients exhibit multiple phenotypes due to differences in clinical features, triggers, airway inflammation, and physiologic and pathologic characteristics. Different airway inflammatory phenotypes distinguish asthma classifications: type 2 inflammatory asthma and non-type 2 inflammatory asthma (90). Asthma is considered to be a classic type 2 inflammatory disease, characterized by the activation of Th2 cells and type 2 intrinsic lymphoid cells (ILC2). IgE, released by type 2 cytokines such as IL-4, IL-13, and IL-5 and plasma cell activation, stimulates basophils, mast cells, and eosinophils, causing epithelial cell activation and airway smooth muscle spasm (91, 92). Non-type 2 asthma is closely associated with a neutrophilic airway inflammatory response. In non-type 2 asthma, pathogens and stimuli activate Th17 and Th1 cells and stimulate neutrophils by releasing IL-6, IL-17, IL-8, IFN-γ, and TNF-α, resulting in inflammation (93). Current therapies primarily focus on suppressing inflammation and relieving obstruction but face limitations in efficacy and side effects, particularly concerning the critical pathological feature of airway remodeling (4).

Numerous studies have revealed a close association between the severity of asthma attacks and IL-37 levels. Lunding et al. (94) and Gao et al. (95) confirmed lower IL-37 expression in peripheral blood mononuclear cells (PBMCs) of pediatric and adult asthmatics compared with healthy controls. This difference became more pronounced following stimulation with CD3/CD28, which mimics the activation of T cells by antigen-presenting cells, suggesting that in asthma patients, the response of PBMCs to stimulation may lead to the downregulation of IL-37 expression, thereby exacerbating inflammatory responses (94). This reduction in expression is not confined to the circulatory system but is also in the local airway microenvironment: Charrad et al. (96) further confirmed the reduced expression levels of IL-37 mRNA in induced sputum and serum of asthma patients, and noted a significant negative correlation between this expression level and the severity of asthma. Additionally, a study from 2021 indicated that reduced serum IL-37 levels were closely associated with worsening asthma conditions, particularly during asthma exacerbations, where levels were significantly lower than in healthy individuals and stable asthma patients, and positively correlated with pulmonary function indicators (FEV1) (97).These findings collectively support the potential key role of IL-37 in the initiation, progression, and persistence of asthma. Synthesizing these findings, reduced IL-37 expression demonstrates a multi-tiered pattern: baseline deficiency, dysregulated suppression upon immune challenge, and dynamic declines in both local (airway) and systemic (serum) levels. This pattern’s significant correlation with asthma severity, exacerbations, and impaired lung function indicates that IL-37 deficiency serves as a key biomarker throughout asthma initiation, progression, and persistence, suggesting its potential functional role in disease pathogenesis.

The core therapeutic potential of IL-37 in asthma stems from its ability to intervene in the pathogenic mechanisms of asthma, including the inhibition of pro-inflammatory cytokine production, the reduction of cytokines that impair airway epithelial barrier function, and the prevention of airway remodeling.

The impact of IL-37 on T cell subsets is particularly noteworthy. It can inhibit the activation and proliferation of Th2 and Th17 cells, which play a central role in the inflammatory response of asthma (98). Th2 cells produce IL-4 and IL-13, key cytokines in asthma inflammation, while Th17 cells produce IL-17, which is closely related to the pathogenesis of neutrophilic and eosinophilic asthma (90). By reducing the production of these pro-inflammatory cytokines, IL-37 helps alleviate airway inflammation (98).

As previously mentioned, the activation of Th2 cells leads to a Th1/Th2 imbalance, resulting in increased production of Th2 cytokines such as IL-4, IL-13, and IL-5, which induce allergen-specific IgE synthesis and the release of inflammatory mediators (89).

In an ovalbumin (OVA)-induced asthma model, Lunding et al. (94) found that intranasal administration of recombinant human IL-37 (rhIL-37) reduced levels of IL-4, IL-5, IL-6, IL-12, and IL-13, thereby attenuating Th2 cell responses. Huang et al. (99) also discovered that rhIL-37 protein significantly lowered IL-4, IL-6, and IL-13 levels, while increasing IFN-γ expression in the OVA-induced asthma model group. Additionally, Cui et al. (83) further confirmed the anti-inflammatory effects of IL-37 in an OVA-induced asthma model, showing that transgenic mice expressing IL-37a and IL-37b had a significant reduction in eosinophils in the lungs, with a minor increase in neutrophils and no significant changes in lymphocyte and macrophage counts compared to wild-type mice.

In a house dust mite (HDM)-induced asthma mouse model, IL-37 has demonstrated significant anti-inflammatory effects. Meng (100) and Zhu (101) found that administration of recombinant human IL-37 protein, either via intranasal inhalation or intravenous injection, effectively reduced levels of IL-4, IL-5, IL-6, and IL-13 in a chronic HDM-induced asthma model. In the study by Zhu et al. (101), NOD/SCID mice were specifically utilized as an HDM-induced asthma model, where the mice were sensitized and challenged with HDM to replicate the pathological processes of allergic asthma. Following the administration of rhIL-37, a decrease in the production of IL-17, CCL2, CCL17, CCL11, and CCL5 in the lungs and bronchoalveolar lavage fluid (BALF) of these experimental mice was observed. These findings underscore the potential of rhIL-37 as a therapeutic agent to alleviate airway inflammation and associated symptoms in asthma models. Although Lv et al. (102) reported that IL-37 did not affect the production of Th2-associated cytokines in an HDM-induced acute asthma model, they observed a significant reduction in eosinophilia, CCL11 production, and airway hyperresponsiveness (AHR) when IL-37 was administered during the challenge phase. Collectively, these studies consistently indicate that IL-37 possesses the potential to mitigate airway inflammation, eosinophil infiltration, and AHR, highlighting its therapeutic potential in the alleviation of asthma symptoms.

IL-17 is a cytokine produced by a specific subset of T cells, namely Th17 cells. Th17 cells are a T cell subset in the immune system that plays a significant role in inflammation and autoimmune diseases. In the pathogenesis of asthma, the activation and proliferation of Th17 cells, and their production of IL-17, particularly IL-17A, are closely associated with the development of neutrophilic and eosinophilic asthma. Charrad et al. (96) found that IL-37 inhibits IL-17A production in CD4+ T cells from the sputum of patients with asthma. IL-24 increased epithelial-derived IL-17A, worsening neutrophilic airway inflammation. In 16-HBE cells, IL-37 decreased IL-24-induced epithelial-derived IL-17A production by modulating p-STAT3 and p-ERK1/2 pathways. In HDM/LPS-sensitized asthmatic mice, *in vivo* rhIL-37 therapy reduced IL-17A levels and Th17 immune response in the lungs (103). IL-37 ameliorated CS-induced lung inflammation in mice and reduced the production of pro-inflammatory cytokines such as IL-1β, IL-6, IL -17, monocyte chemoattractant protein-1, and TNF-α (84). This underscores the significant role of IL-37 in inhibiting Th17 cell-mediated inflammation.

The involvement of the airway epithelium in the pathophysiology of asthma is becoming more widely acknowledged. When the epithelial barrier function is disrupted, the inflammatory response of the airway epithelium to specific triggers (e.g., allergens) and nonspecific triggers (e.g., viruses, or smoke) is exacerbated and is accompanied by an increased release of TSLP, IL-33, and IL -25 (88, 104). Secretion of these epithelial factors activates several immune cells, including Th2 and ILC2 cells. In addition, TSLP and IL-33 directly activate mast cells, thereby establishing a direct link between airway epithelial and mast cell activation without the involvement of T2 cells, leading to airway inflammation and airway hyperresponsiveness (105). Thus, TSLP and IL-33 play a key role in immune hyperresponsiveness that mediates asthma attacks. According to Berraes et al. (106) in 2016, the application of rhIL-37 inhibited the production of TSLP in sputum cells from asthma patients, in which IL-37’s suppressive effect on TSLP was similarly expressed in isolated bronchial epithelial cells. In 2019, Meng et al. (100) found that IL-37 intranasally suppressed TSLP expression in the airway epithelial cells of mice with chronic allergic asthma caused by HDM. *In vitro*, IL-37 inhibited NF-κB and ERK1/2 activation in human bronchial epithelial cells to reduce HDM-induced TSLP expression. In 2024, Wang et al. (107) further demonstrated that rhIL-37 significantly decreased IL-33 levels in BALF of HDM-induced asthmatic mice and diminished IL-33 secretion in HDM-stimulated 16HBE cells; the same study showed IL-37 preserves barrier function by inhibiting store-operated calcium entry (SOCE). Thus, as an upstream regulator, IL-37 represents an innovative and alternative therapeutic strategy to reduce airway TSLP and IL-33 levels. Compared with healthy controls, asthma patients exhibit a substantial increase in IL-1β and IL-33 to IL-37 expression/production ratio. IL-37 alleviates allergic airway inflammation by balancing the disease-amplifying effects of IL-1β and IL-33 (45). Furthermore, because of its regulatory role in allergic inflammation, IL-37 is a vital element in maintaining local immunological homeostasis.

**Figure 2:**
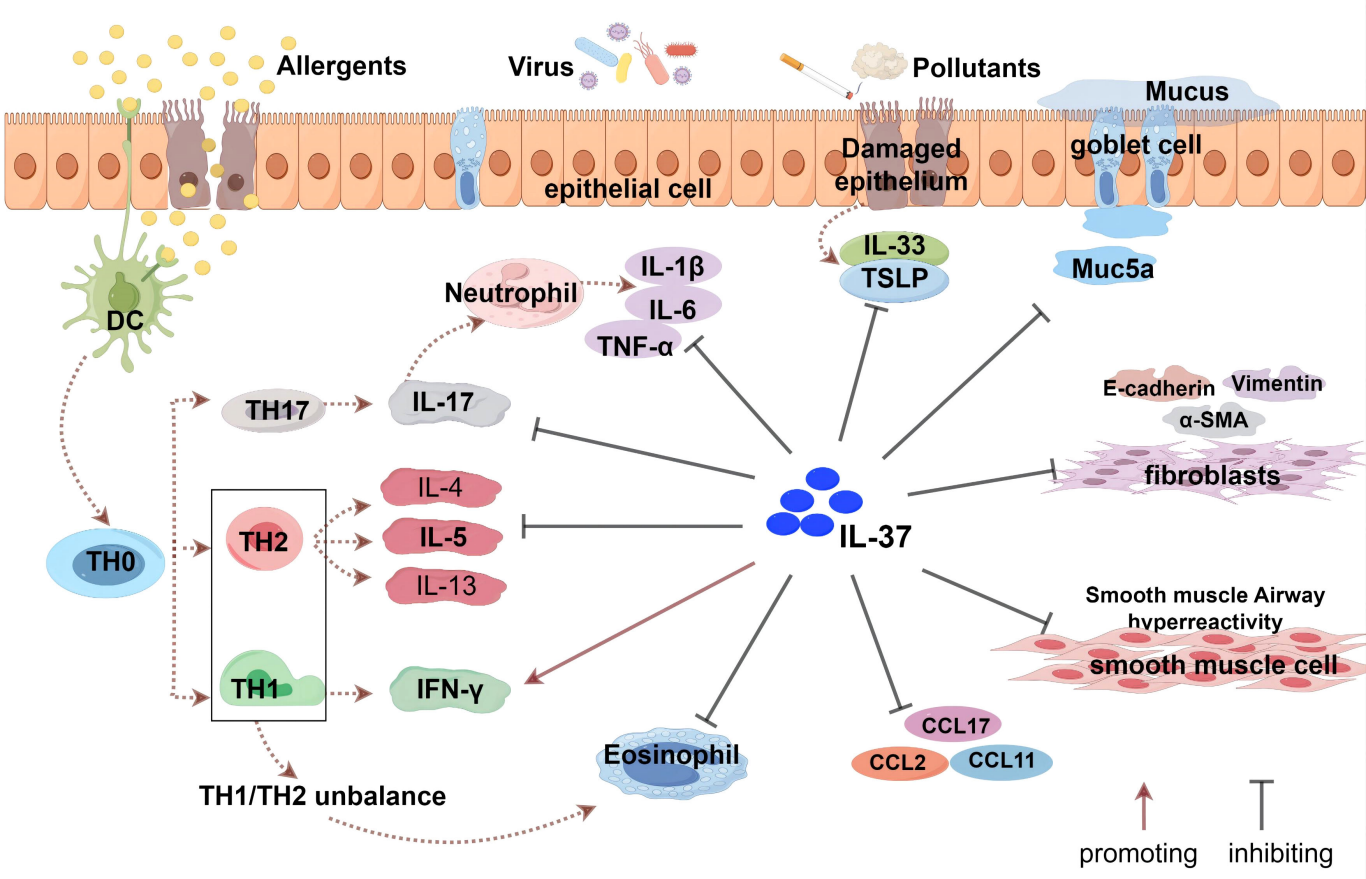
The role of IL-37 in asthma. IL-37 indirectly enhances the levels of IFN-γ by regulating the balance between Th1 and Th2 cells, but does not directly activate Th1 cells. It inhibits the responses of Th2 and Th17 cells and suppresses the production of related cytokines (such as IL-4, IL-5, IL-17, etc.) and eosinophil chemoattractant protein (CCL11). Additionally, IL-37 affects bronchial epithelial cells, airway smooth muscle cells, and fibroblasts, modulating the secretion of related cytokines and chemokines, including TSLP and IL-33 produced by epithelial cells. IL-37 can also inhibit the epithelial-mesenchymal transition (EMT) process, which is associated with the thickening of the airway wall and subepithelial fibrosis.

Airway remodeling is characterized by epithelial barrier failure, goblet cell metaplasia, thickening of the airway smooth muscle layer, and angiogenesis. All these characteristics promote steroid-resistant asthma and acute exacerbations of asthma. Therefore, new therapeutic strategies are needed to replace steroid hormones. Huang et al. (99) discovered that IL-37 administration effectively suppressed TGF-β1-induced cell proliferation, migration, epithelial-mesenchymal transition (EMT), and the inflammatory response of airway smooth muscle cells in a mouse model of allergic airway inflammation in asthma. Furthermore, by reducing NF-κB STAT3 activation, IL-37 reduced airway inflammation and reformed asthma. According to Feng et al. (103), IL-37 prevents airway remodeling by reversing bronchial EMT in chronic asthma via the IL-24 signaling pathway. Beyond these direct effects on core remodeling mechanisms, IL-37 also shows potential in mitigating the impact of environmental triggers. Wang et al. (108) discovered that IL-37 reduced airway hyperresponsiveness in particulate matter 2.5 (PM2.5)-exposed mice and decreased aberrant cell contraction, proliferation, and migration in human amniotic mesenchymal stromal cells cultured with PM2.5. These findings could pave the way for the development of IL-37 as a therapeutic agent, as well as identifying pharmacological targets for preventing and treating asthma airway remodeling (**Figure 2**).

IL-37, as an anti-inflammatory cytokine, shows great promise in the treatment of asthma. It not only suppresses the production of various pro-inflammatory cytokines to alleviate airway inflammation but also regulates the function of airway epithelial cells, inhibiting the release of epithelial factors such as TSLP. These effects suggest that IL-37 could become an effective therapeutic intervention for alleviating asthma symptoms, improving lung function, and controlling disease progression, particularly in cases involving steroid resistance and remodeling. Future research should further explore the application of IL-37 in asthma treatment and how to effectively integrate it into existing treatment plans to provide better quality of life and disease control for asthma patients.

### 3.2 Pulmonary infection

#### 3.2.1 Corona viruses

To date, three highly pathogenic human coronaviruses (CoVs) have been identified: Severe Acute Respiratory Syndrome Coronavirus (SARS-CoV), Middle East Respiratory Syndrome Coronavirus (MERS-CoV), and Severe Acute Respiratory Syndrome Coronavirus 2 (SARS-CoV-2). However, as no studies have been reported on the association between IL-37 and SARS-CoV, this section focuses on the role of IL-37 in infections caused by MERS-CoV and SARS-CoV-2.

The COVID-19 pandemic induced by SARS-CoV-2 is a public health crisis. More evidence links COVID-19 to cytokine release syndrome, specifically elevated pro-inflammatory cytokines (IL-1β, IL-6, TNF-α, and IL-1α) in extreme cases (47, 107). Inflammation-associated pro-inflammatory cytokines in COVID-19, particularly the IL-1 family, may be suppressed by the anti-inflammatory cytokine IL-37 (109). Li (110) discovered a link between elevated plasma IL-37 response levels and clinical regression profiles such as shorter length of hospitalization, faster viral nucleic acid negative conversion, earlier CT imaging improvement, faster resolution of cough symptoms, and better benign prognosis in patients with early-stage COVID-19. Notably, higher IL-37 plasma levels lowered IL-6, IL-8, and ultrasensitive C-reactive protein but did not affect type I interferon plasma levels. As a result, IL-37 may not impair type I interferon’s anti-viral activity while exerting its anti-inflammatory function, which is a definite advantage over the hormonal anti-inflammatory scheme now employed in clinical practice. Following SARS-CoV-2 infection, IL-37 substantially inhibited the inflammatory response of human angiotensin-converting enzyme 2 transgenic mice. Early IL-37 injection reduces lung tissue injury and inflammatory cell infiltration, promoting disease remission (110). For the Omicron variant, IL-37 specifically inhibits macrophage-driven hyperinflammation by suppressing NF-κB activation (111). A recent study found that IL-37 expression was reduced in the serum of individuals with severe COVID-19 (112). This downregulation may be associated with an increased risk of developing this disease. Furthermore, a strong connection was found between IL-37 gene mutations and an increased frequency of severe COVID-19 (113). Data demonstrate that IL-37 may have a protective role in SARS-CoV-2-induced inflammatory consequences. Thus, low IL-37 plasma levels can be used to predict the severity of COVID-19 (**Table 1**).

**Table 1:** The role of IL-37 and its biological functions in pulmonary infectious diseases.

Disorder	Mode	Tissue/cell type	Results/Role of IL-37	References
COVID-19	Human	Plasma	- Increased il-37 levels accompany a favorable prognosis (patients with COVID-19 presented significantly higher plasma IL-37 levels (mean [SEM], 196.2 [35.78] pg/mL)) - Reduce IL-6,IL-8 and CRP levels	(110)
	Mouse	lung tissue	- attenuate lung inflammation and alleviate respiratory tissue damage	
	Human	serum	- IL-37 was down-regulated in serum of patients with severe COVID-19 (109.2 VS. 125.4 ng/L; p <0.001)	(112)
	Human	gene	- two variants of IL37 gene (rs3811046 and rs3811047) may be associated with susceptibility to COVID-19 (TG genotype of SNP rs3811046 showed a significantly increased frequency in patients compared to controls (61.0 VS. 38.0%; OR = 2.55; 95% CI = 1.45-4.50; p = 0.002). GA genotype of SNP rs3811047 also showed an increased frequency in patients compared to controls (39.0 VS. 24.0%; OR = 2.02; 95% CI = 1.10-3.71; p = 0.033; pc = 0.165)	(117)
Influenza A virus (IAV)	Human	Serum	- The IL-37 levels in the sera and PBMCs of patients infected with IAV were higher than those of healthy subjects (324.4 + 43.17 pg/mL vs 129.2 ± 19.23 pg/mL, P < 0.05)	(117)
	Vitro	A549 cells PBMCs	- The expression of IL-37 mRNA and protein in IAV-infected A549 cells and PBMCs was upregulated, and IL-37 protein was able to inhibit the replication of IAV RNA	
Tuberculosis (TB)	Human	serum	- IL-37 was significantly elevated in patients with TB	(119) (122) (120)
	Human	serum	- Coexpression of serum IL-18BP and IL-37 is a more discriminative biomarker for diagnostic active PTB than the single monitoring serum IL-18BP	(121)
	Human	serum	- Stimulates IL-10 (p < 0.0001) and TGF-B poduction (p = 0.0011) - Downregulates IL-12 (p = 0.0116) and IFN-r (p < 0.01) production	(119)
	Vitro	macrophages	- inducing macrophages towards an M2-like phenotype	(119)
	Human	plasma	- Inhibits IL-1B,IL-6 and TNF-a production	(120)
	Human	gene	- IL-37 gene polymorphisms associated with susceptibility of TB	(123) (124) (125)
	Mouse	spleen	- Regulated T cell responses, elevated TH1 cells, and decreased TH17 cells	(123)
Streptococcus pneumoniae	Mouse	lung tissue	- Decreases -1B,IL-6 and TNF-a production from RAW macrophages - Inhibits kiling capacity of RAW macrophages	(129)
aspergillosis	Mouse	lung tissue	- Reduces NLRP3 inftammasome actiwity - Inhibits IL-1β secretion	(131)
Candida infections	Mouse	lung tissue	- reduce the production of proinflammatory cytokines - suppresse neutrophil recruitment	(132)

Infection with the Middle East Respiratory Syndrome Coronavirus (MERS-CoV) is associated with a mortality rate exceeding 35% (114). This high fatality rate may be attributed to rapid viral replication and excessive activation of pro-inflammatory cytokines and chemokines. Similar to COVID-19, patients with severe MERS exhibit not only elevated respiratory viral loads but also significantly increased serum concentrations of cytokines such as IL-6, interferon-α (IFN-α), and IP-10 (115). Using an animal model, Qi et al. (114) demonstrated that treatment with IL-37 resulted in reduced viral load, decreased levels of the pulmonary chemokine MCP-1, and lower serum concentrations of MCP-1, IFN-γ, IL-17A, IL-6, and IL-10 compared to the control group. In contrast, mRNA expression of the anti-inflammatory cytokines IL-10 and IL-20 was up-regulated in lung tissue. Consistent results were obtained in cellular models, where IL-37 treatment led to reduced viral titers and significantly lower levels of inflammatory factors, including IL-6 and TNF-α.

In summary, IL-37 exerts anti-inflammatory effects in both MERS-CoV and SARS-CoV-2 infections by inhibiting the secretion of pro-inflammatory cytokines. However, the underlying mechanisms require further systematic and comprehensive investigation. Future studies should explore whether IL-37 has analogous anti-inflammatory roles in other respiratory viral infections, thereby providing potential clinical strategies for mitigating inflammation induced by respiratory viral infections.

#### 3.2.2 Influenza

Influenza virus infection, particularly those caused by the Influenza A virus (IAV), poses a significant global public health challenge. IAV is known to cause acute upper respiratory tract infections and can spread rapidly through airborne transmission, leading to regional and seasonal epidemics and even global pandemics. The activation of the host immune system during influenza infection triggers a cascade of inflammatory responses and complications, posing a severe threat to human health (116).

Expressed in a variety of cell types and tissues, IL-37 has garnered considerable attention for its potential role in influenza virus infection. Studies have shown that IAV infection induces a significant upregulation of IL-37 expression in patient sera and peripheral blood mononuclear cells (PBMCs) (85). This upregulation may reflect a role for IL-37 in inhibiting IAV replication, as demonstrated *in vitro* experimental studies. Increased expression of IL-37 in A549 cells and PBMCs infected with IAV, along with the ability of recombinant human IL-37 to suppress IAV RNA replication and reduce viral titers, highlight the direct antiviral effects of IL-37. These findings provide a scientific basis for considering IL-37 as a therapeutic strategy against IAV infection. Compared to other anti-inflammatory cytokines such as IL-10, IL-37 exhibits unique antiviral properties, particularly in its ability to inhibit viral replication and mitigate inflammation. The therapeutic potential of IL-37 extends beyond its antiviral effects to its capacity to modulate immune responses. IL-37 has the potential to alleviate inflammation caused by influenza infection, reduce tissue damage, and improve outcomes by modulating macrophage function and MAPK signaling pathways (117).

Translating IL-37 into clinical treatment for influenza involves addressing challenges such as optimizing dosage, selecting appropriate administration routes, and determining the best timing for treatment. Additionally, it is necessary to ensure its effectiveness against various strains of the flu and to confirm its safety for patients with strong immune responses. IL-37 shows promise in treating influenza by reducing inflammation and regulating immune responses. However, further research is needed to fully understand its mechanisms of action and potential clinical applications. It has the potential to revolutionize the treatment of influenza by aiding in viral control, potentially enhancing the effects of vaccines, and contributing to the development of new immunotherapy strategies(**Table 1**).

#### 3.2.3 Tuberculosis

Tuberculosis (TB) is the leading cause of death from infectious diseases globally, especially in developing and least-developed countries. The most frequently afflicted organ in a TB infection is the lung. Pulmonary TB (PTB) is correlated with higher rates of mortality and morbidity. Early detection of PTB is critical for lowering mortality (118). In patients with active tuberculosis (ATB), serum levels of IL-37 are elevated. A 2015 study indicated that prior to a six-month course of standard anti-TB drug therapy with rifampicin, isoniazid, pyrazinamide, and ethambutol, ATB patients had significantly higher serum IL-37 levels and mRNA expression compared to post-treatment patients or healthy individuals (119). Similarly, Zhang et al. (120) discovered that individuals with ATB had significantly higher levels of IL-37 plasma than healthy controls and that IL-37 plasma levels were lowered after antituberculosis medication. Several follow-up investigations verified the considerable elevation of IL-37 levels in patients with PTB. Wawrocki et al. (121) observed that co-expression of serum IL-37 and IL-18BP was a more discriminating biomarker for the diagnosis of active PTB than serum IL-18BP alone. Furthermore, Yu et al. (122) observed overexpressed IL-37, TNF-ɑ, and miR-155 levels in elderly patients with active PTB. The simultaneous assessment of serum IL-37/TNF-ɑ/miR-155 expression holds promise as a diagnostic modality for identifying active PTB in older individuals. These data imply that IL-37 plays an important role in the course of PTB. IL-37 levels are considered an indicator of recovery and a new biomarker for the identification and diagnosis of ATB.

In addition to serum elevated IL-37 levels, the single nucleotide polymorphisms (SNP) of the IL-37 gene may affect susceptibility to TB or IL-37 levels. Liu et al. (123) discovered a correlation between IL-37 gene polymorphisms and susceptibility to TB. The proportion of C/C genotypes was significantly higher in Saudi patients with ATB for rs2723176 (-6962 A/C), and this SNP’s C allele was correlated with patients with TB (124). In addition, the C allele of the rs2723176 SNP was associated with elevated levels of circulating IL-37. Regardless of gender, age, or clinical disease type, IL-37 levels were elevated in the serum of patients with TB, according to an Iraqi study (125). SNPs in the promoter region of the IL-37 gene are believed to influence susceptibility or resistance to TB infection. These findings suggest that IL-37 regulates the TB inflammatory response.

The initial line of defense against Mycobacterium tuberculosis entering the lungs is composed of macrophages, DCs, and granulocytes. To regulate pathogen transmission, human macrophages phagocytose M. tuberculosis and attract peripheral blood lymphocytes, or monocytes, at the site of infection. M. tuberculosis has evolved numerous strategies for manipulating macrophage polarization (M1/M2) to evade the host’s immunological response (126). In this context, IL-37 plays a significant role. Huang et al. discovered that IL-37 inhibits the phagocytic activity and NO production of macrophages after stimulation by iH37Rv, and promotes the differentiation of macrophages towards an M2 phenotype. Regarding the source of IL-37 in tuberculosis, Huang et al. (127) found that mannose-encapsulated lipid arabinomannan (ManLAM, M. tuberculosis’s primary cell wall component and virulence factor) promoted IL-37 production in human type II alveolar epithelial cells via the TLR2/p38 or ERK1/2 pathways. In addition, M. tuberculosis induced the production of IL-37 in macrophages, according to Liu et al. (123). Meanwhile, IL-37 also has an impact on the inflammatory environment produced by M. tuberculosis. Huang et al. (119) discovered a negative correlation between serum IL-37 levels and serum IFN-γ and IL-12 concentrations and a positive correlation between serum IL-10 and TGF-β1 levels in patients with ATB. Zhang et al. (120) found IL-37 production is inversely correlated with the immune response to pro-inflammatory cytokines such as IL-1β, IL-6, and TNF-ɑ and associated with prolonged or complicated TB as well as greater TB burden. IL-37 production not only lowered pro-inflammatory cytokines produced by M. tuberculosis infection but also decreased Th17 cell responses to control the growing inflammation. IL-37 also boosted adaptive immunity and increased Th1 cells, both of which are required for M. tuberculosis containment in an IFN γ-dependent manner. Taken together, the anti-inflammatory properties of IL-37 may represent a unique molecular therapeutic target for the treatment of M. tuberculosis (**Table 1**).

#### 3.2.4 Streptococcus pneumoniae infection

Streptococcus pneumoniae (Spn), a Gram-positive coccus, poses a significant threat to human health as a crucial respiratory pathogen. In the course of pneumococcal pneumonia, excessive immune activation and tissue damage can facilitate bacterial invasion, a critical factor leading to severe complications. Therefore, limiting proinflammatory cytokine responses and leukocyte influx at appropriate times is crucial for ensuring the proper resolution of inflammation (128).

However, the anti-inflammatory effect of IL-37 may also have negative implications for the host’s immune defense. A study by Schauer et al. (129) found that compared to RAW vector cells, RAW macrophages stably transfected with human IL-37 exhibited a 70% decrease in the production of cytokines such as IL-6, TNF-α, and IL-1β, and a 2.2-fold reduction in intracellular killing ability against Streptococcus pneumoniae. This suggests that while IL-37 has significant anti-inflammatory effects, it may also affect the host’s ability to clear pathogens.

In a mouse transgenic model expressing human IL-37b (IL-37tg), researchers observed that IL-37 reduced the expression of cytokines IL-6, TNF-α, and IL-1β during the early stages of Streptococcus pneumoniae infection. However, in later stages, as bacterial loads increased and bacteremia spread, the expression levels of these cytokines in lung tissue rose, accompanied by a significant increase in the recruitment of alveolar macrophages and neutrophils. Additionally, TRAIL mRNA expression decreased by three-fold (129). This altered immune response led to the development of necrotic pneumonia and increased mortality, revealing that the anti-inflammatory properties of IL-37 may, to some extent, weaken the mice’s ability to control Streptococcus pneumoniae infection in the lungs.

The balance between pro- and anti-inflammatory cytokines during Streptococcus pneumoniae infection is crucial for effective pathogen clearance and preventing lung damage. Future therapies should aim to enhance pathogen elimination while minimizing tissue inflammation. Understanding IL-37 and immune regulation is key to developing strategies that protect against excessive inflammation during infection. This may include modulating IL-37 expression and activity to ensure a host response that clears pathogens without causing undue harm (**Table 1**).

#### 3.2.5 Fungal infection

Fungal pneumonia occurs primarily in immunocompromised individuals. Impairment of neutrophils and other myeloid cells is critical for the development of invasive fungal infections (Aspergillus, the causative agent of mucormycosis). In contrast, impairment of T cell function and granulomatous inflammatory processes is critical for susceptibility to Cryptococcus, Pneumocystis, and type II fungi. Fungal infection induces inflammatory chemical mediators, cytokines, and chemokines released by mast cells (130). In a mouse model of aspergillosis, IL-37 significantly reduced NLRP3-dependent neutrophil recruitment and IL-1b secretion, attenuating lung inflammation and injury. As a fundamental inhibitor of innate immunity, IL-37 protects against lung injury by inhibiting pro-inflammatory cytokine production in mice with aspergillosis (131). However, in a Candida albicans infection model (132), IL-37 significantly reduced Candida-induced macrophage inflammatory cytokine production and decreased neutrophil recruitment to Candida. These processes lead to an impaired early innate immune response, which is essential for limiting fungal transmission. Thus, in a mouse model of disseminated candidiasis, overexpression of IL-37 is detrimental to early host defenses against Candida albicans. In patients with mycetoma, serum levels of IL-1β and IL-12 positively correlate with lesion size and disease duration, whereas IL-35 and IL-37 show a negative correlation. Consistent with the Candida model, elevated IL-37 in these patients suppresses macrophage-derived cytokines, including IL-1β and IL-12, thereby dampening innate immunity and facilitating disease progression (133). The differences in the anti-inflammatory effects of IL-37 exhibited in these three different disease models are primarily related to an imbalance in inflammatory regulation. Therefore, the balance between effector immune responses and tissue immune damage should be tightly regulated to prevent clinical complications of the disease (**Table 1**). Further studies are warranted to elucidate the mechanisms by which IL-37 influences the outcomes of fungal infections. This will not only enhance our understanding of its role in disease pathogenesis but also potentially position IL-37 as a therapeutic target for preventing disease onset and progression.

### 3.3 Association between IL-37 and NSCLC

Globally, the incidence and mortality rates of cancer are on the rise, with lung cancer being the most common and deadliest form, imposing a significant burden on society and the economy. NSCLC accounts for 80-85% of lung cancer cases and is the primary subtype of the disease (134, 135). Recent interest in the role of interleukin-37 (IL-37) in NSCLC progression has emerged, with studies suggesting its potential to exert protective effects through various mechanisms, offering new avenues for lung cancer therapy (136).

IL-37 expression in NSCLC tissues is closely associated with the progression of the tumor. Research by Ge et al. (82) has shown that IL-37 mRNA and protein levels are significantly reduced in NSCLC tissues compared to normal tissue, with low expression correlating with advanced tumor stages and higher TNM stages. Jiang et al. (137) further confirmed this phenomenon, finding that serum IL-37 levels in NSCLC patients are lower than those in healthy controls, significantly associated with advanced TNM stages. These findings suggest that low IL-37 expression may indicate the deterioration of NSCLC, although its precise biological significance and clinical implications warrant further investigation.

IL-37 has a direct inhibitory effect on NSCLC tumor cells. Studies have demonstrated that exogenous IL-37 can induce apoptosis in human lung adenocarcinoma A549 cells, while inhibiting their proliferation, migration, and invasion (138). Additionally, co-transfection of IL-37 with CCL22 can further reduce the proliferation rate of A549 cells and inhibit the epithelial-mesenchymal transition (EMT) process (139). IL-37 also directly inhibits tumor cell invasion and migration by suppressing the IL-6/STAT3 signaling pathway and its downstream targets, including Bcl-2, NEDD9, and cyclin D1 (137).

In terms of immune regulation, IL-37 enhances anti-tumor immune responses by suppressing the chemotactic nature of Tregs. Tumors frequently evade immune surveillance by elevating the local Treg population, and IL-37 can attenuate the accumulation of Tregs within the tumor microenvironment, thereby weakening their protective shield for cancer cells (140). Chen et al. (139) validated *in vitro* that IL-37 significantly curbs tumor growth in a transplanted A549 lung adenocarcinoma model, and further experiments demonstrated its capacity to diminish the chemotactic properties of Tregs. Additionally, IL-37 indirectly impedes tumor cell migration by modulating the Rac1 signaling pathway. Rac1, a member of the Rho GTPase family, plays a pivotal role in the development of various malignancies. Recent findings suggest that the intracellular mature form of IL-37 can block the activation of Rac1 and its downstream signaling by binding to the Rac1 CAAX motif, thus suppressing the migratory behavior of lung cancer cells (141). Furthermore, reduced IL-37 expression in human lung adenocarcinoma biopsy samples is associated with tumor metastasis, indicating its potential crucial role in preventing the dissemination of tumors. IL-37 also exerts anti-tumor effects by regulating the m6A modification activity of lung cancer cells and inhibiting the Wnt5a/5b pathway. These mechanisms collectively reshape gene expression patterns and signal transduction pathways, hindering the malignant transformation of lung cancer cells (142).

**Figure 3:**
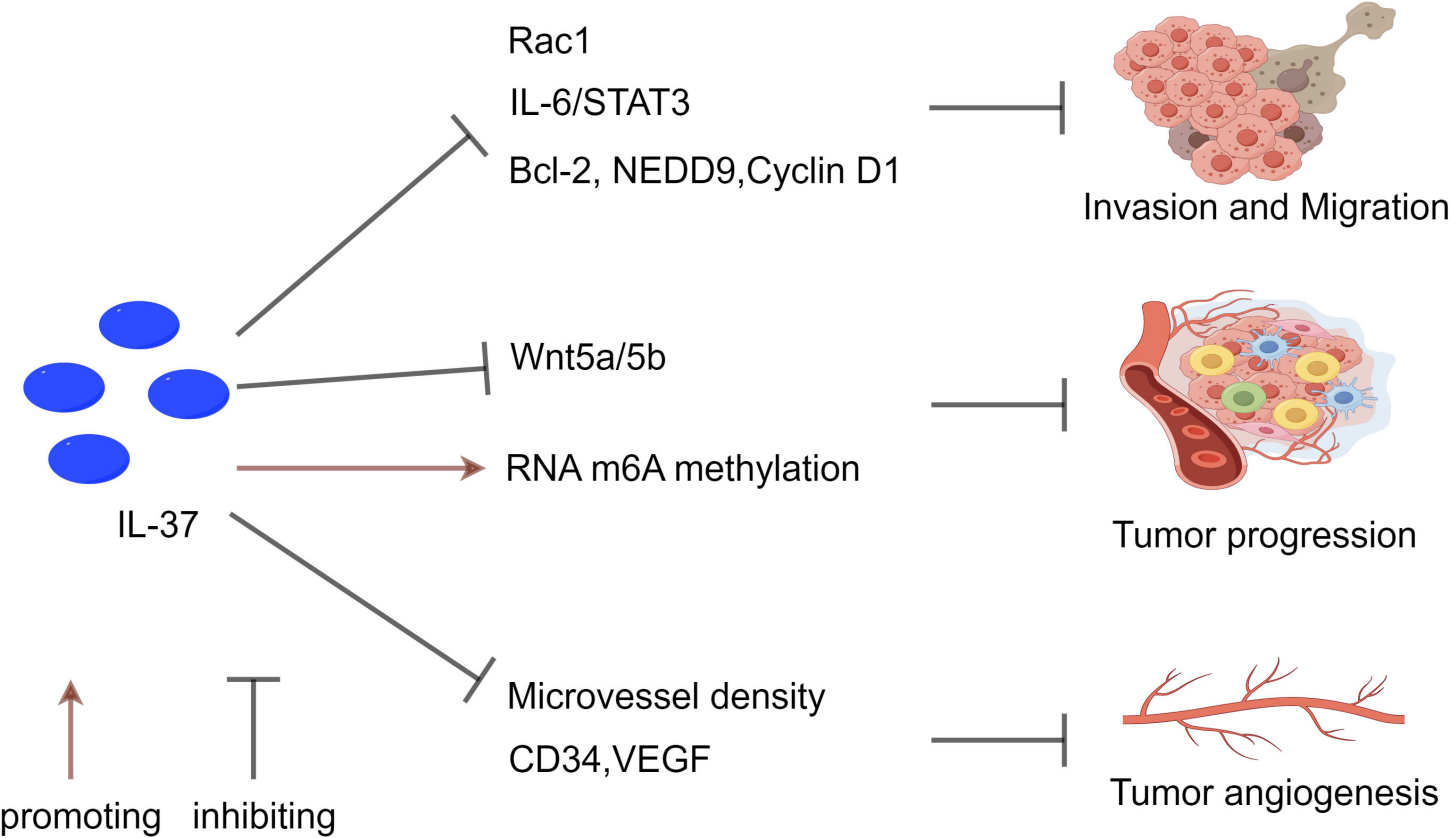
The inhibitory effect of IL-37 on NSCLC. IL-37 suppresses tumor metastasis and invasion through multiple pathways. It inhibits Rac1 activation and the IL-6/STAT3 pathway, reducing downstream targets Bcl-2, NEDD9, and Cyclin D1. Moreover, IL-37 influences tumor progression by modulating RNA m6A methylation in T lymphocytes, while also inhibiting the Wnt5a/5b pathway and pro-apoptotic protein expression. Additionally, IL-37 contributes to the regulation of tumor angiogenesis by decreasing levels of CD34 and VEGF, which are associated with tumor angiogenesis. The reduction in IL-37 expression correlates with high microvascular density (MVD), emphasizing its significance in tumor angiogenesis, metastasis, and progression.

Given the multiple protective effects displayed by IL-37 in NSCLC, future research should delve into its molecular mechanisms and expression and function across different types of lung cancer, as well as consider its anti-tumor activity and potential immunomodulatory effects in the development of novel therapeutic strategies (**Figure 3**).

### 3.4 Pulmonary interstitial fibrosis

Pulmonary fibrosis is a chronic inflammatory disease characterized by excessive collagen deposition. There are currently no viable treatments for pulmonary fibrosis. IL-37 is a recently identified anti-inflammatory cytokine. Topical IL-37 therapy reduced bleomycin (BLM)-induced experimental lung inflammation and fibrosis, according to Li et al. (86). In BLM-induced C57BL/6 mice, lentivirus-producing IL-37 was administered intranasally. IL-37 increased mouse survival and decreased the weight loss caused by BLM. Additionally, in the lungs of the BLM-treated mice, IL-37 decreased hydroxyproline levels and collagen deposition as well as inflammatory infiltrates. Finally, in lung tissue from BLM-stimulated animals, IL-37 administration decreased the expression of monocyte chemoattractant protein-1, IL-6, and TNF-ɑ but enhanced the expression of IFN-ɣ. Similarly, a recent study found that patients with idiopathic pulmonary fibrosis (IPF) have reduced lung IL-37 expression. In contrast, IL-37 inhibited BLM-induced fibrosis/lung injury in experiments. Antifibrotic effects of IL-37 are achieved by suppression of the TGF-β1 signaling pathway and promotion of autophagy. According to research by Kim et al. (87), macrophages and alveolar epithelial cells (AECs) in IPF patients exhibited significantly less IL-37 protein than healthy controls. In mice, IL-37 dose-dependently reduced oxidative stress-induced primary AEC mortality. However, inhibiting IL-37 significantly enhanced the death of AEC (A549 cells) derived from human lung cancer. In primary human lung fibroblasts, IL-37 suppressed the synthesis of constitutive fibronectin and type I collagen mRNAs and proteins. IL-37 inhibited TGF-β1-induced lung fibroblast proliferation and disrupted the TGF-β1 signaling pathway. Moreover, in IPF fibroblasts, IL-37 enhanced beclin-1-dependent autophagy and autophagy regulators. BLM injection-induced inflammation and collagen deposition were dramatically decreased by IL-37. At the moment, the precise regulation mechanism of IL-37 against interstitial fibrosis is unknown. Nonetheless, these results imply that IL-37 may be a viable therapeutic option for fibrotic lung disorders.

## 4 Conclusion

In summary, accumulating evidence suggests that aberrant levels of Interleukin-37 (IL-37) are extensively involved in the pathogenesis of various respiratory diseases. As an anti-inflammatory cytokine with immunomodulatory functions, IL-37 demonstrates broad application prospects in the treatment of respiratory disorders. Specifically, IL-37 exerts protective effects in diseases such as asthma, pulmonary infections, NSCLC, and pulmonary fibrosis through core mechanisms including inhibition of pro-inflammatory cytokine release, modulation of immune cell functions, regulation of autophagy and apoptosis, as well as suppression of fibrotic formation. However, this anti-inflammatory property of IL-37 may, to a certain extent, weaken the ability of mice to control pneumococcal infection in the lungs. The expression level of IL-37 is closely associated with the severity of respiratory diseases, indicating its potential as a biomarker for diagnosis and prognostic evaluation. Furthermore, IL-37 significantly inhibits tumor cell proliferation, metastasis, and angiogenesis, highlighting its value in NSCLC immunotherapy.

Although significant progress has been made, several important questions remain to be explored in depth. Future research should focus on systematically elucidating the precise molecular mechanisms and signaling networks of IL-37 across different cell types and disease models. Additionally, novel delivery strategies are required to enhance the stability and efficacy of IL-37-based therapeutic regimens. At the preclinical stage, as dose-effect responses have emerged in the current research on IL-37 in type 2 diabetes, it is crucial to comprehensively evaluate the dose-response relationship and safety of recombinant IL-37 under various pathological conditions in the future. Moreover, large-scale multicenter cohort studies are necessary to validate the clinical utility of IL-37 as a diagnostic or prognostic biomarker.

In conclusion, IL-37 is not only a critical immunoregulatory factor but also a promising diagnostic tool and therapeutic target in the field of respiratory diseases. Integrating multidisciplinary approaches from immunology, molecular biology, and clinical medicine will facilitate the translation of IL-37-related discoveries into clinical practice, providing novel strategies for the prevention and treatment of respiratory diseases, and ultimately improving patient outcomes.
